# Supplementary material for: Predicting sex, age, general cognition and mental health with machine learning on brain structural connectomes
Source: Hum Brain Mapp. 2022 Dec 21;44(5):1913–33. doi: 10.1002/hbm.26182 (PMC9980898; doi:10.1002/hbm.26182)
Supplement: Supplementary file 1 — Data S1: Supporting Information [file HBM-44-1913-s001.pdf]

Predicting sex, age, general cognition and mental health with machine learning on brain structural connectomes
Supplementary Materials

Hon Wah Yeung[1], Aleks Stolicyn[1], Colin R. Buchanan[2,9,10], Elliot M. Tucker-Drob[3,4], Mark E. Bastin[5,9,10], Saturnino Luz[6], Andrew M. McIntosh[1,7], Heather C. Whalley[1,*], Simon R. Cox[2,9,10,*], and Keith Smith[8,*]

[1]Department of Psychiatry, University of Edinburgh, Edinburgh, United Kingdom
[2]Department of Psychology, University of Edinburgh, Edinburgh, United Kingdom
[3]Department of Psychology, University of Texas, Austin, TX, USA
[4]Population Research Center and Center on Aging and Population Sciences, University of Texas at Austin, TX, USA
[5]Centre for Clinical Brain Science, University of Edinburgh, Edinburgh, United Kingdom
[6]Usher Institute, Edinburgh Medical School, The University of Edinburgh, Edinburgh, United Kingdom
[7]Centre for Genomic and Experimental Medicine, Institute of Genetics and Molecular Medicine, University of Edinburgh, Edinburgh, UK
[8]Department of Physics and Mathematics, Nottingham Trent University, Nottingham, United Kingdom
[9]Lothian Birth Cohorts, University of Edinburgh, Edinburgh, United Kingdom
[10]Scottish Imaging Network, A Platform for Scientific Excellence Collaboration (SINAPSE), Edinburgh, United Kingdom
[*]These authors share joint senior authorship

November 4, 2022

A Materials and Methods

A.1 UK Biobank Field ID

The UK Biobank field IDs of the cognitive tasks and mental health questionnaire items used in this study were shown below.

A.1.1 Cognitive tasks

Verbal Numerical Reasoning (UKB Field ID: 20016.2.0):

Participants were asked to answer 13 multiple-choice questions and the score is the number of questions answered correctly in two minutes.

Reaction Time Task (UKB Field ID: 20023.2.0):

Two cards were shown on the screen in front of the participants in each trial. They were asked to press the button-box as quickly as possible when the two cards have matching symbols. There were 12 trials and the first 5 were for training. 4 out of the remaining 7 trials have matching cards. The score is the mean time to to press the button-box for these 4 trials.

Pairs Matching (UKB Field ID: 399.2.2):

This task aims at testing the participants' visual memory and the scores were obtained from the second trial. Participants were asked to memorise the patterns 6 pairs of cards. The cards were then faced down and participants needed to correctly identify the pairs of cards with matching patterns. The score was the number of errors made before matching all pairs.

Prospective Memory (UKB Field ID: 20018.2.0):

At the start of the UK Biobank cognitive test battery, participants would see this instruction on the screen "At the end of the games we will show you four coloured symbols and ask you to touch the Blue Square. However, to test your memory, we want you to actually touch the Orange Circle instead.". After completing the cognitive tasks, participants were asked to touch the Blue Square. Participants who correctly touched the orange circle on first attempt were coded 1 and 0 otherwise in this study.

A.1.2 Mental Health Questionnaire

Field ID	Description
20401.0.0	Ever addicted to any substance or behaviour
20421.0.0	Ever felt worried, tense, or anxious for most of a month or longer
20425.0.0	Ever worried more than most people would in similar situation
20441.0.0	Ever had prolonged loss of interest in normal activities
20446.0.0	Ever had prolonged feelings of sadness or depression
20463.0.0	Ever heard an un-real voice
20468.0.0	Ever believed in an un-real conspiracy against self
20471.0.0	Ever seen an un-real vision
20474.0.0	Ever believed in un-real communications or signs
20480.0.0	Ever self-harmed
20499.0.0	Ever sought or received professional help for mental distress
20500.0.0	Ever suffered mental distress preventing usual activities
20501.0.0	Ever had period of mania / excitability
20502.0.0	Ever had period extreme irritability

A.2 Principal Component Analysis

A.2.1 Comparison between using Pearson's and polychoric correlation

Figure 1 showing the scree plot. For our dataset, the MHQ-factor derived with Pearson's correlation explained 28.03% for the variance, while the MHQ-factor derived with polychoric correlation explained 49.60% for the variance. Th scree plots for both methods indicated that the significant dimensions were spanned by the first 3 eigenvectors, where the eigenvalues were larger than one. The congruence coefficients for the loadings between the two methods of the first three principal components (PC) were 0.9428, 0.9406 and 0.9686 respectively, and the correlation for the first three principal component scores between the two methods were 0.9748, 0.8866 and 0.9738 respectively. As such, it is clear that both methods for deriving the MHQ-factor convey highly similar information. As such, we present results based on polychoric correlation method for the MHQ-factor in all subsequent analyses, as it explained a significantly larger variance.

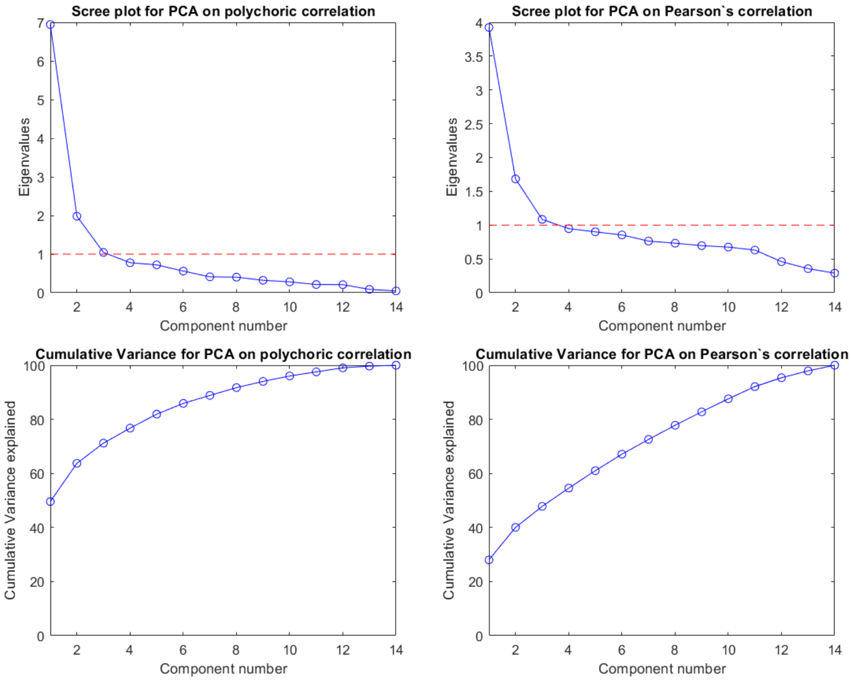

Figure 1: The scree plots and cumulative variance explained for PCA with Pearson's correlation and PCA with polychoric correlation respectively

A.2.2 Numeric loadings for PCA with polychoric correlation

Table 1: The principal component loadings on the Mental Health Questionnaire items

Loadings	PC1	PC2	PC3	PC4	PC5	PC6	PC7	PC8	PC9	PC10	PC11	PC12	PC13	PC14
20401.0.0	0.500	0.001	0.489	-0.281	0.638	0.102	-0.060	0.041	0.078	-0.033	0.038	0.005	0.003	-0.009
20421.0.0	0.789	-0.292	-0.154	0.056	-0.005	0.322	-0.194	-0.002	-0.183	0.129	0.045	0.258	0.075	0.019
20425.0.0	0.787	-0.294	0.086	0.065	-0.078	0.353	-0.013	-0.143	-0.171	-0.131	-0.161	-0.237	-0.047	-0.021
20441.0.0	0.815	-0.360	-0.106	0.062	0.052	-0.286	-0.225	0.099	0.063	-0.005	-0.070	-0.132	0.064	0.126
20446.0.0	0.799	-0.376	-0.243	0.053	0.028	-0.235	-0.235	0.028	0.087	-0.085	0.031	0.060	-0.119	-0.116
20463.0.0	0.616	0.613	-0.205	0.014	0.162	-0.058	-0.016	0.014	-0.096	0.377	-0.076	-0.109	-0.062	-0.020
20468.0.0	0.762	0.432	0.023	0.064	-0.199	0.219	-0.064	0.072	0.138	-0.047	0.308	-0.076	-0.083	0.049
20471.0.0	0.569	0.496	-0.367	0.175	0.300	-0.146	0.145	-0.169	-0.188	-0.247	0.063	0.041	0.037	0.016
20474.0.0	0.632	0.565	-0.202	-0.204	-0.126	0.151	-0.049	-0.015	0.301	-0.094	-0.216	0.069	0.077	-0.018
20480.0.0	0.639	0.040	0.221	-0.539	-0.280	-0.233	-0.012	-0.315	-0.113	0.030	0.058	0.030	-0.001	0.011
20499.0.0	0.785	-0.383	-0.097	-0.080	0.023	0.016	0.404	0.078	0.076	0.028	-0.085	0.129	-0.130	0.061
20500.0.0	0.846	-0.336	-0.093	-0.033	-0.045	-0.027	0.269	0.103	0.058	0.079	0.137	-0.123	0.164	-0.076
20501.0.0	0.629	0.381	0.423	0.061	-0.209	-0.141	0.016	0.384	-0.210	-0.099	-0.079	0.063	0.008	-0.021
20502.0.0	0.580	0.049	0.484	0.552	-0.047	-0.100	0.043	-0.269	0.147	0.083	-0.034	0.071	0.020	-0.007

B Results from deep learning and classical machine learning methods

B.1 Abbreviations in the circular plots

Abbreviation	Full Name	Abbreviation	Full Name
ctx	Cortex	lingual	lingual
lh	left hemisphere	mOFC	medial orbito frontal
rh	right hemisphere	MTG	middle temporal
THA	thalamus proper	ParaHG	parahippocampal
CAU	caudate	ParaCL	paracentral
PUT	putamen	ParsOp	pars opercularis
PAL	pallidum	ParsOrb	pars orbitalis
brain.stem	brain stem	ParsTr	pars triangularis
HIP	hippocampus	PCalCC	perical carine
AMYG	amygdala	PoCG	postcentral
Accumbens	accumbens area	PCC	posterior cingulate
VentralDC	ventral diencephalon	PrCG	precentral
banksSTS	banks of superior temporal sulcus	precuneus	precuneus
cACC	caudal anterior cingulate	rACC	rostral anterior cingulate
cMFG	caudal middle frontal	rMFG	rostral middle frontal
cuneus	cuneus	SFG	superior frontal
entorhinal	entorhinal	SPL	superior parietal
fusiform	fusiform	STG	superior temporal
IPL	inferior parietal	SMG	supramarginal
ITG	inferior temporal	FPC	frontal pole
ICgG	isthmus cingulate	TPC	temporal pole
lOC	lateral occipital	TTG	transverse temporal
lOFC	lateral orbito frontal	insula	insula

B.2 BrainNetCNN hyperparameter tuning and results

B.2.1 Model Hyperparameter Tuning

The BrainNetCNN models follow the layer order: E2E, E2N, N2G and a fully connected layer. Leaky ReLU activation and dropout were added after E2E, E2N and N2G layers. After taking out the test set, the rest of the data was split into training and validation sets with a ratio of 4:1 (this split is for hyperparameter tuning only and not used in the main analysis). Bayesian optimization was used to tune the hyperparameters within user-specified ranges of values based on the validation set. We tuned the number of filters for E2E (4 - 64 filters), E2N (4 - 128 filters), N2G (4 - 256 filters), dropout rate (0 – 0.95), Leaky ReLU scale (0 - 1), learning rate (1e-4 - 1e-2), L2 regularisation (1e-10 - 1e-4).

Table 2: Hyperparameters for the BrainNetCNN model. Under "Model Structure", the numbers represent the number of filters or node for each layer, with all the model following the same layer order: E2E, E2N, N2G and a fully connected layer

**Prediction Task**	**Model Structure**	**Dropout rate**	**Leaky ReLU scale**	**Learning rate**	**L2 regularisation**	**Optimizer**
Sex	22, 126, 240, 2	8.25 e-5	0.0089	0.0015	3.37e-9	Adam
Age	33, 125, 229, 1	0.0072	0.0573	0.0017	1.60e-10	Adam
g-factor	10, 95, 247, 1	1.43e-5	0.0383	0.0011	1.72e-6	Adam
MHQ-factor	10, 103, 186, 1	0.0021	3.38e-5	0.0010	1.18e-8	Adam

B.2.2 Results with hyperparameter tuning implemented

**Weights**	MD	FA	SC	OD	ISOVF	ICVF
**Validation**	79.07 (0.72)	82.22 (0.63)	89.14 (1.28)	82.57 (1.41)	82.34 (0.85)	81.22 (0.68)
**Training**	90.74 (3.27)	93.14 (3.57)	96.77 (2.93)	93.88 (3.76)	98.42 (3.16)	94.31 (3.99)
**Test**	77.90 (0.42)	81.43 (0.64)	86.81 (0.82)	81.82 (1.17)	82.86 (0.88)	79.46 (1.29)

(a) Sex prediction accuracies (mean percentage with standard deviation in brackets) with BrainNetCNN model for different connectivity weightings

**Weights**	MD	FA	SC	OD	ISOVF	ICVF
**Validation**	4.610 (0.662)	4.646 (0.664)	4.146 (0.731)	4.345 (0.705)	4.290 (0.717)	4.644 (0.645)
**Training**	3.495 (0.825)	3.728 (0.806)	3.523 (0.814)	3.561 (0.820)	3.445 (0.832)	3.950 (0.768)
**Test**	4.670 (0.645)	4.725 (0.655)	4.179 (0.711)	4.533 (0.668)	4.446 (0.684)	4.617 (0.648)

(b) Age prediction performance (mean absolute errors with correlations in brackets) with BrainNetCNN model for different connectivity weightings

**Weights**	MD	FA	SC	OD	ISOVF	ICVF
**Validation**	0.234 (0.757)	0.235 (0.754)	0.253 (0.752)	0.236 (0.757)	0.237 (0.759)	0.237 (0.757)
**Training**	0.271 (0.750)	0.264 (0.750)	0.312 (0.741)	0.290 (0.744)	0.270 (0.751)	0.282 (0.747)
**Test**	0.208 (0.767)	0.223 (0.765)	0.222 (0.769)	0.213 (0.768)	0.22 (0.766)	0.218 (0.767)

(c) *g*-factor prediction performance (correlations with mean absolute error in brackets) with BrainNetCNN model with age and sex covariates for different connectivity weightings

**Weights**	MD	FA	SC	OD	ISOVF	ICVF
**Validation**	0.224 (0.760)	0.226 (0.764)	0.227 (0.762)	0.227 (0.762)	0.225 (0.764)	0.227 (0.767)
**Training**	0.247 (0.756)	0.244 (0.761)	0.258 (0.755)	0.241 (0.760)	0.246 (0.760)	0.254 (0.761)
**Test**	0.248 (0.764)	0.246 (0.769)	0.240 (0.768)	0.243 (0.768)	0.239 (0.769)	0.244 (0.772)

(d) MHQ-factor prediction performance (correlations with mean absolute error in brackets) with BrainNetCNN model with age and sex covariates for different connectivity weightings

Table 3: Prediction performances with BrainNetCNN model of four different prediction tasks based on different connectivity weightings

B.3 Prediction performances of different classical machine learning models

B.3.1 Linear Ridge model

**Weights**	MD	FA	SC	OD	ISOVF	ICVF
**Validation**	80.91 (0.43)	84.15 (0.88)	89.67 (1.04)	85.20 (0.63)	84.12 (0.58)	83.89 (0.45)
**Training**	93.79 (5.03)	94.43 (0.43)	93.75 (1.40)	92.94 (2.75)	91.52 (2.09)	97.80 (2.69)
**Test**	80.91 (0.55)	84.44 (0.32)	87.54 (0.48)	84.52 (0.40)	84.68 (0.59)	83.38 (0.46)

(a) Sex prediction accuracies (mean percentage with standard deviation in brackets) with Linear Ridge model for different connectivity weightings

**Weights**	MD	FA	SC	OD	ISOVF	ICVF
**Validation**	4.472 (0.680)	4.481 (0.679)	4.221 (0.713)	4.422 (0.688)	4.204 (0.712)	4.549 (0.668)
**Training**	3.103 (0.855)	2.968 (0.866)	3.773 (0.779)	3.668 (0.795)	3.711 (0.789)	3.085 (0.856)
**Test**	4.465 (0.683)	4.624 (0.661)	4.258 (0.698)	4.534 (0.665)	4.250 (0.696)	4.408 (0.693)

(b) Age prediction performance (mean absolute errors with correlations in brackets) with Linear Ridge model for different connectivity weightings

**Weights**	MD	FA	SC	OD	ISOVF	ICVF
**Validation**	0.255 (0.753)	0.256 (0.754)	0.270 (0.748)	0.267 (0.750)	0.259 (0.751)	0.256 (0.753)
**Training**	0.318 (0.743)	0.323 (0.744)	0.330 (0.735)	0.347 (0.734)	0.330 (0.735)	0.328 (0.742)
**Test**	0.253 (0.761)	0.262 (0.762)	0.248 (0.764)	0.254 (0.759)	0.249 (0.759)	0.258 (0.762)

(c) g-factor prediction performance (correlations with mean absolute error in brackets) with Linear Ridge model with age and sex covariates for different connectivity weightings

**Weights**	MD	FA	SC	OD	ISOVF	ICVF
**Validation**	0.220 (0.770)	0.216 (0.771)	0.223 (0.767)	0.225 (0.768)	0.223 (0.769)	0.220 (0.772)
**Training**	0.320 (0.752)	0.339 (0.749)	0.281 (0.753)	0.318 (0.749)	0.299 (0.753)	0.336 (0.75)
**Test**	0.244 (0.777)	0.240 (0.779)	0.244 (0.772)	0.247 (0.774)	0.241 (0.775)	0.242 (0.779)

(d) MHQ-factor prediction performance (correlations with mean absolute error in brackets) with Linear Ridge model with age and sex covariates for different connectivity weightings

Table 4: Prediction performances with Linear Ridge model of four different prediction tasks based on different connectivity weightings

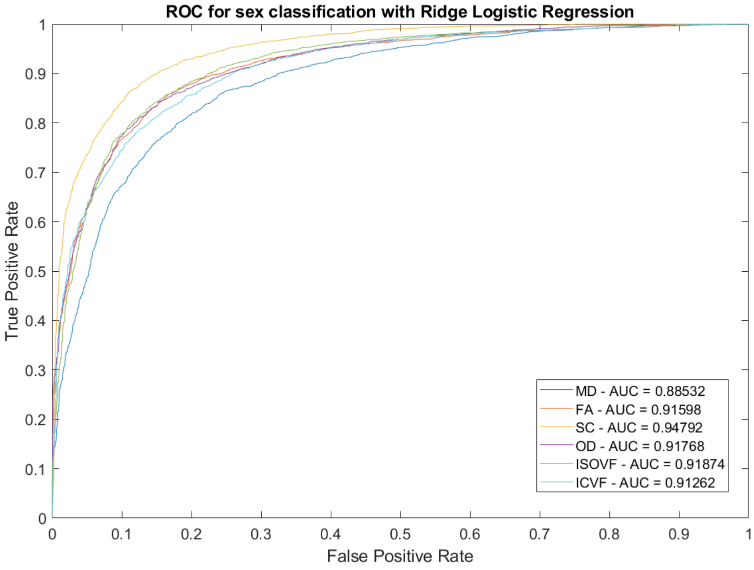

Figure 2: AUC and ROC curve for sex classification with Ridge Logistic Regression

B.3.2 Linear LASSO model

Weights	MD	FA	SC	OD	ISOVF	ICVF
**Validation**	79.97 (0.50)	82.81 (0.94)	88.22 (1.42)	85.36 (0.60)	84.24 (0.34)	81.79 (0.63)
**Training**	86.62 (1.01)	90.30 (1.03)	90.41 (0.29)	92.07 (0.78)	91.88 (1.28)	90.26 (1.86)
**Test**	79.80 (0.68)	82.70 (0.57)	85.90 (0.68)	84.76 (0.53)	84.65 (0.25)	80.80 (0.97)

(a) Sex prediction accuracies (mean percentage with standard deviation in brackets) with Linear LASSO model for different connectivity weightings

Weights	MD	FA	SC	OD	ISOVF	ICVF
**Validation**	4.459 (0.678)	4.326 (0.693)	4.238 (0.710)	4.374 (0.709)	4.224 (0.709)	4.469 (0.667)
**Training**	3.936 (0.774)	3.652 (0.805)	3.897 (0.763)	3.550 (0.795)	3.708 (0.788)	3.766 (0.792)
**Test**	4.456 (0.671)	4.382 (0.680)	4.282 (0.695)	4.571 (0.680)	4.274 (0.693)	4.384 (0.681)

(b) Age prediction performance (mean absolute errors with correlations in brackets) with Linear LASSO model for different connectivity weightings

Weights	MD	FA	SC	OD	ISOVF	ICVF
**Validation**	0.241 (0.756)	0.223 (0.761)	0.268 (0.749)	0.256 (0.754)	0.252 (0.752)	0.237 (0.756)
**Training**	0.339 (0.733)	0.364 (0.725)	0.335 (0.733)	0.357 (0.728)	0.312 (0.740)	0.321 (0.737)
**Test**	0.243 (0.760)	0.241 (0.762)	0.248 (0.765)	0.244 (0.761)	0.240 (0.761)	0.242 (0.761)

(c) g-factor prediction performance (correlations with mean absolute error in brackets) with Linear LASSO model with age and sex covariates for different connectivity weightings

Weights	MD	FA	SC	OD	ISOVF	ICVF
**Validation**	0.223 (0.769)	0.211 (0.772)	0.226 (0.768)	0.214 (0.769)	0.228 (0.768)	0.219 (0.769)
**Training**	0.267 (0.758)	0.328 (0.739)	0.279 (0.755)	0.312 (0.744)	0.275 (0.756)	0.308 (0.746)
**Test**	0.240 (0.775)	0.224 (0.781)	0.237 (0.776)	0.230 (0.779)	0.247 (0.773)	0.235 (0.777)

(d) MHQ-factor prediction performance (correlations with mean absolute error in brackets) with Linear Ridge model with age and sex covariates for different connectivity weightings

Table 5: Prediction performances with Linear LASSO model of four different prediction tasks based on different connectivity weightings

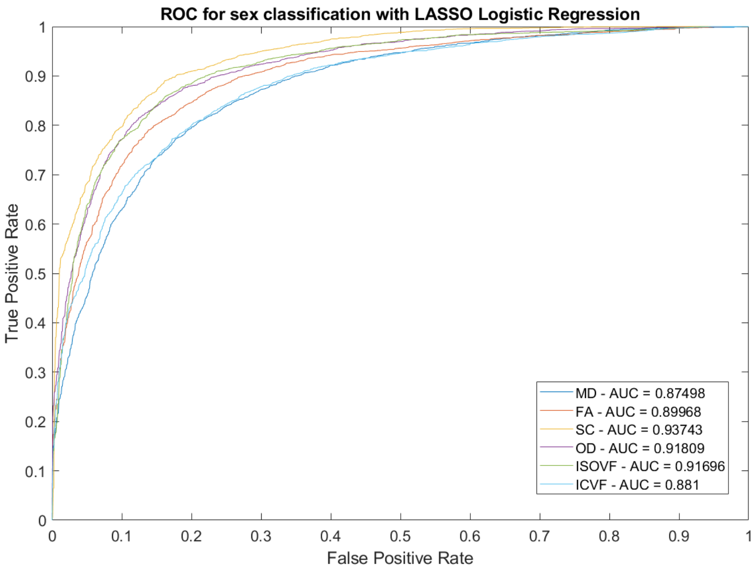

Figure 3: AUC and ROC curve for sex classification with LASSO Logistic Regression

B.3.3 Linear SVM model

Weights	MD	FA	SC	OD	ISOVF	ICVF
**Validation**	82.05 (0.80)	84.63 (0.82)	89.77 (0.91)	85.83 (0.43)	84.37 (0.65)	85.55 (1.13)
**Training**	96.05 (1.27)	94.06 (2.46)	93.90 (0.89)	92.06 (1.37)	92.15 (1.50)	98.33 (0.51)
**Test**	80.65 (0.62)	83.56 (1.12)	87.27 (0.56)	84.68 (0.35)	84.18 (0.52)	84.37 (0.82)

(a) Sex prediction accuracies (mean percentage with standard deviation in brackets) with Linear SVM model for different connectivity weightings

Weights	MD	FA	SC	OD	ISOVF	ICVF
**Validation**	4.574 (0.660)	4.450 (0.680)	4.318 (0.698)	4.428 (0.683)	4.369 (0.690)	4.614 (0.654)
**Training**	3.214 (0.806)	3.009 (0.827)	3.975 (0.738)	3.156 (0.816)	3.147 (0.817)	3.167 (0.81)
**Test**	4.537 (0.664)	4.649 (0.656)	4.357 (0.684)	4.527 (0.662)	4.453 (0.679)	4.500 (0.672)

(b) Age prediction performance (mean absolute errors with correlations in brackets) with Linear SVM model for different connectivity weightings

Weights	MD	FA	SC	OD	ISOVF	ICVF
**Validation**	0.257 (0.752)	0.256 (0.752)	0.270 (0.745)	0.268 (0.748)	0.260 (0.748)	0.257 (0.752)
**Training**	0.311 (0.738)	0.313 (0.740)	0.316 (0.730)	0.330 (0.729)	0.322 (0.729)	0.319 (0.737)
**Test**	0.257 (0.761)	0.264 (0.762)	0.246 (0.763)	0.247 (0.760) 0.250 (0.759)	0.261 (0.762)	

(c) *g*-factor prediction performance (correlations with mean absolute error in brackets) with Linear SVM model with age and sex covariates for different connectivity weightings

Weights	MD	FA	SC	OD	ISOVF	ICVF
**Validation**	0.218 (0.743)	0.215 (0.744)	0.224 (0.794)	0.226 (0.740)	0.221 (0.743)	0.216 (0.744)
**Training**	0.299 (0.717)	0.304 (0.717)	0.286 (0.750)	0.309 (0.711)	0.272 (0.724)	0.306 (0.716)
**Test**	0.245 (0.745)	0.245 (0.746)	0.230 (0.794)	0.248 (0.743)	0.240 (0.745)	0.244 (0.747)

(d) MHQ-factor prediction performance (correlations with mean absolute error in brackets) with Linear SVM model with age and sex covariates for different connectivity weightings

Table 6: Prediction performances with Linear SVM model of four different prediction tasks based on different connectivity weightings

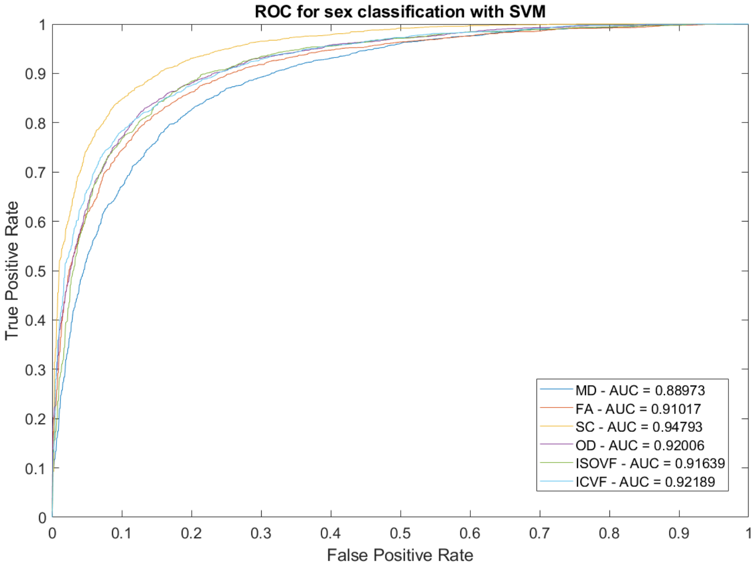

Figure 4: AUC and ROC curve for sex classification with SVM

B.3.4 Kernel Ridge model

Weights	MD	FA	SC	OD	ISOVF	ICVF
**Validation**	79.62 (0.45)	81.62 (0.85)	86.44 (1.30)	83.30 (0.71)	82.53 (0.65)	80.50 (0.96)
**Training**	90.46 (0.12)	91.16 (0.23)	88.09 (0.36)	91.56 (0.80)	89.94 (0.42)	91.29 (0.59)
**Test**	79.50 (0.66)	80.86 (0.22)	84.28 (0.36)	82.89 (0.46)	82.73 (0.43)	79.84 (0.76)

(a) Sex prediction accuracies (mean percentage with standard deviation in brackets) with Kernel Ridge model for different connectivity weightings

Weights	MD	FA	SC	OD	ISOVF	ICVF
**Validation**	4.550 (0.653)	4.550 (0.651)	4.402 (0.685)	4.313 (0.693)	4.244 (0.707)	4.655 (0.632)
**Training**	3.856 (0.766)	3.902 (0.761)	4.271 (0.707)	3.761 (0.778)	3.768 (0.778)	3.990 (0.746)
**Test**	4.485 (0.655)	4.491 (0.660)	4.434 (0.669)	4.378 (0.671)	4.255 (0.693)	4.563 (0.642)

(b) Age prediction performance (mean absolute errors with correlations in brackets) with Kernel Ridge model for different connectivity weightings

Weights	MD	FA	SC	OD	ISOVF	ICVF
**Validation**	0.251 (0.753)	0.234 (0.769)	0.245 (0.759)	0.188 (0.804)	0.178 (0.801)	0.241 (0.767)
**Training**	0.307 (0.741)	0.337 (0.731)	0.354 (0.728)	0.463 (0.690)	0.450 (0.699)	0.344 (0.728)
**Test**	0.252 (0.759)	0.241 (0.773)	0.257 (0.768)	0.191 (0.804)	0.176 (0.806)	0.241 (0.773)

(c) *g*-factor prediction performance (correlations with mean absolute error in brackets) with Kernel Ridge model with age and sex covariates for different connectivity weightings

Weights	MD	FA	SC	OD	ISOVF	ICVF
**Validation**	0.220 (0.769)	0.208 (0.785)	0.207 (0.774)	0.188 (0.789)	0.218 (0.774)	0.220 (0.769)
**Training**	0.274 (0.757)	0.301 (0.743)	0.269 (0.758)	0.352 (0.725)	0.303 (0.744)	0.284 (0.754)
**Test**	0.245 (0.775)	0.226 (0.793)	0.229 (0.780)	0.207 (0.797)	0.218 (0.790)	0.245 (0.774)

(d) MHQ-factor prediction performance (correlations with mean absolute error in brackets) with Kernel Ridge model with age and sex covariates for different connectivity weightings

Table 7: Prediction performances with Kernel Ridge model of four different prediction tasks based on different connectivity weightings

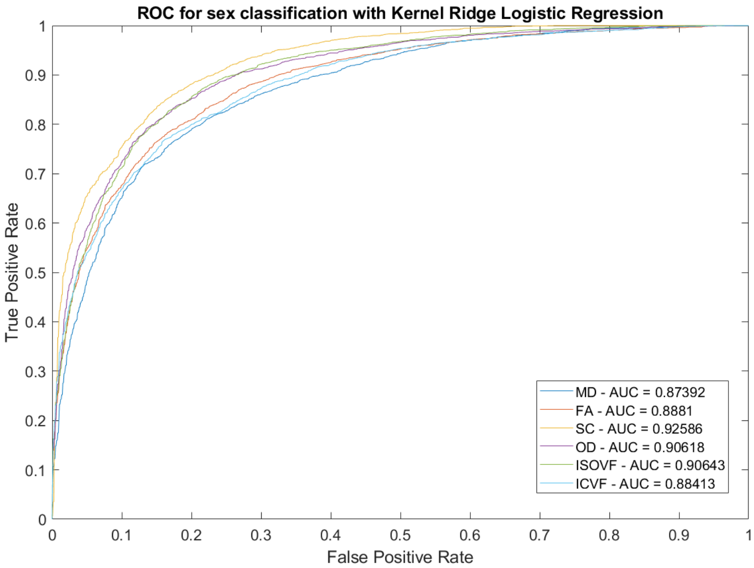

Figure 5: AUC and ROC curve for sex classification with Kernel Ridge Logistic Regression

B.4 Consistency for the gradient attribution maps and beta coefficients

B.4.1 Consistency across folds

Table 8: The consistency for the gradient attribution maps across folds for BrainNetCNN and the consistency for the beta coefficients across folds for linear ridge regressions of different prediction tasks based on streamline count, illustrated using Spearman's correlation

	Streamline Count Structural Connectivity									
	BrainNetCNN					Linear Ridge Regression				
Sex Classification		CV1	CV2	CV3	CV4		CV1	CV2	CV3	CV4
	CV2	0.803	0.799	0.798	0.802	CV2	0.840	0.827	0.861	0.782
	CV3		0.801	0.792	0.803	CV3		0.859	0.857	0.719
	CV4			0.815	0.799	CV4			0.867	0.705
	CV5				0.812	CV5				0.736
Age Prediction		CV1	CV2	CV3	CV4		CV1	CV2	CV3	CV4
	CV2	0.748	0.760	0.754	0.762	CV2	0.816	0.810	0.823	0.827
	CV3		0.762	0.760	0.753	CV3		0.827	0.830	0.830
	CV4			0.776	0.760	CV4			0.832	0.826
	CV5				0.773	CV5				0.841
g-factor Prediction		CV1	CV2	CV3	CV4		CV1	CV2	CV3	CV4
	CV2	0.586	0.580	0.559	0.581	CV2	0.721	0.703	0.696	0.714
	CV3		0.579	0.581	0.603	CV3		0.706	0.673	0.709
	CV4			0.563	0.563	CV4			0.672	0.692
	CV5				0.559	CV5				0.667
MHQ-factor Prediction		CV1	CV2	CV3	CV4		CV1	CV2	CV3	CV4
	CV2	0.528	0.516	0.510	0.485	CV2	0.676	0.665	0.681	0.652
	CV3		0.555	0.560	0.530	CV3		0.691	0.710	0.689
	CV4			0.537	0.497	CV4			0.693	0.657
	CV5				0.502	CV5				0.692

Table 9: The consistency for the gradient attribution maps across folds for BrainNetCNN and the consistency for the beta coefficients across folds for linear ridge regressions of different prediction tasks based on fractional anisotropy, illustrated using Spearman's correlation

	Fractional Anisotropy Structural Connectivity									
	BrainNetCNN					Linear Ridge Regression				
Sex Classification		CV1	CV2	CV3	CV4		CV1	CV2	CV3	CV4
	CV2	0.831	0.821	0.811	0.823	CV2	0.872	0.863	0.867	0.873
	CV3		0.819	0.831	0.833	CV3		0.854	0.862	0.872
	CV4			0.820	0.824	CV4			0.859	0.863
	CV5				0.818	CV5				0.863
Age Prediction		CV1	CV2	CV3	CV4		CV1	CV2	CV3	CV4
	CV2	0.832	0.818	0.818	0.833	CV2	0.716	0.709	0.731	0.737
	CV3		0.807	0.813	0.834	CV3		0.718	0.714	0.734
	CV4			0.808	0.819	CV4			0.705	0.719
	CV5				0.807	CV5				0.740
g-factor Prediction		CV1	CV2	CV3	CV4		CV1	CV2	CV3	CV4
	CV2	0.648	0.605	0.603	0.622	CV2	0.763	0.749	0.720	0.742
	CV3		0.630	0.616	0.638	CV3		0.760	0.759	0.786
	CV4			0.601	0.624	CV4			0.752	0.751
	CV5				0.617	CV5				0.747
MHQ-factor Prediction		CV1	CV2	CV3	CV4		CV1	CV2	CV3	CV4
	CV2	0.592	0.571	0.598	0.558	CV2	0.684	0.656	0.711	0.664
	CV3		0.593	0.638	0.598	CV3		0.691	0.708	0.698
	CV4			0.608	0.566	CV4			0.708	0.685
	CV5				0.595	CV5				0.699

B.4.2 Consistency across methods

Table 10: The consistency between the gradient attribution maps from BrainNetCNN and the beta coefficients from classical methods of different prediction tasks based on streamline count and fractional anisotropy, illustrated using Spearman's correlation

Connectivity Weight		Streamline Count				Fractional Anisotropy		
Prediction Type	Method	Ridge Regression	LASSO Regression	SVM	Method	Ridge Regression	LASSO Regression	SVM
Sex Classification	CV1	0.736	0.660	0.696	CV1	0.852	0.868	0.781
	CV2	0.770	0.699	0.736	CV2	0.848	0.865	0.812
	CV3	0.750	0.653	0.723	CV3	0.834	0.830	0.774
	CV4	0.759	0.677	0.729	CV4	0.848	0.862	0.837
	CV5	0.637	0.607	0.613	CV5	0.826	0.833	0.809
Age Prediction	CV1	0.708	0.741	0.668	CV1	0.509	0.825	0.630
	CV2	0.710	0.745	0.682	CV2	0.522	0.769	0.623
	CV3	0.682	0.735	0.647	CV3	0.490	0.847	0.618
	CV4	0.699	0.741	0.671	CV4	0.521	0.847	0.632
	CV5	0.713	0.745	0.670	CV5	0.511	0.830	0.627
g-factor Prediction	CV1	0.676	0.455	0.535	CV1	0.775	0.786	0.596
	CV2	0.695	0.531	0.559	CV2	0.781	0.798	0.620
	CV3	0.679	0.491	0.534	CV3	0.776	0.588	0.606
	CV4	0.674	0.515	0.533	CV4	0.788	0.771	0.631
	CV5	0.682	0.468	0.545	CV5	0.780	0.805	0.609
MHQ-factor Prediction	CV1	0.693	0.502	0.496	CV1	0.763	0.787	0.574
	CV2	0.677	0.527	0.545	CV2	0.773	0.792	0.569
	CV3	0.671	0.521	0.523	CV3	0.769	0.809	0.563
	CV4	0.661	0.572	0.486	CV4	0.771	0.794	0.571
	CV5	0.648	0.523	0.511	CV5	0.772	0.801	0.568

B.4.3 Top regions for different prediction tasks of BrainNetCNN and Ridge Regression

Table 11: Top 20 predictive regions based on streamline count and fractional anisotropy connectivities for four different prediction tasks, selected by BrainNetCNN models and linear ridge regression models. Overlapping regions are highlighted in bold text

Connectivity Weight	Streamline Count		Fractional Anisotropy	
Top Regions	BrainNetCNN	Ridge Regression	BrainNetCNN	Ridge Regression
Sex Classification	**ctx-lh-precuneus**	**Right-Putamen**	**Right-Thalamus-Proper**	**Right-Thalamus-Proper**
	**ctx-rh-superiortemporal**	**Left-Putamen**	**Right-Caudate**	**Right-Putamen**
	**ctx-rh-precuneus**	ctx-rh-superiorfrontal	**Right-Putamen**	ctx-lh-precuneus
	**ctx-rh-insula**	**Left-Thalamus-Proper**	**ctx-lh-isthmuscingulate**	**ctx-lh-isthmuscingulate**
	**Right-Putamen**	**Right-Thalamus-Proper**	**ctx-lh-superiorfrontal**	**Left-Putamen**
	**Right-Thalamus-Proper**	**ctx-lh-superiorfrontal**	ctx-lh-rostralmiddlefrontal	**ctx-rh-superiorparietal**
	**ctx-rh-inferiortemporal**	**ctx-rh-precuneus**	**ctx-rh-insula**	**Right-Caudate**
	ctx-lh-supramarginal	**ctx-lh-precuneus**	ctx-rh-posteriorcingulate	**Right-VentralDC**
	ctx-rh-paracentral	ctx-rh-superiorparietal	**ctx-rh-inferiortemporal**	**ctx-rh-inferiortemporal**
	Right-Hippocampus	**ctx-rh-insula**	**Left-Putamen**	ctx-rh-postcentral
	**ctx-rh-supramarginal**	Left-Caudate	Left-Hippocampus	**Left-Thalamus-Proper**
	**Right-VentralDC**	ctx-lh-inferiortemporal	ctx-rh-isthmuscingulate	**ctx-rh-insula**
	**Left-Putamen**	**ctx-lh-isthmuscingulate**	**Right-VentralDC**	**ctx-lh-superiorfrontal**
	**Left-Thalamus-Proper**	**ctx-rh-inferiortemporal**	**ctx-rh-superiorparietal**	**Left-Caudate**
	**ctx-lh-superiortemporal**	**ctx-rh-superiortemporal**	**ctx-rh-precuneus**	**ctx-rh-precuneus**
	ctx-lh-medialorbitofrontal	**ctx-rh-supramarginal**	**Left-Caudate**	ctx-rh-precentral
	**ctx-lh-superiorfrontal**	ctx-lh-superiorparietal	**ctx-rh-superiorfrontal**	ctx-lh-superiorparietal
	**ctx-lh-isthmuscingulate**	**ctx-lh-superiortemporal**	ctx-rh-rostralmiddlefrontal	**ctx-rh-superiorfrontal**
	Right-Accumbens-area	ctx-rh-middletemporal	ctx-rh-lateralorbitofrontal	ctx-lh-inferiorparietal
	ctx-rh-temporalpole	**Right-VentralDC**	**Left-Thalamus-Proper**	ctx-rh-lateraloccipital

Table 11: Top 20 predictive regions based on streamline count and fractional anisotropy connectivities for four different prediction tasks, selected by BrainNetCNN models and linear ridge regression models. Overlapping regions are highlighted in bold text (cont.)

Connectivity Weight	Streamline Count		Fractional Anisotropy	
Top Regions	BrainNetCNN	Ridge Regression	BrainNetCNN	Ridge Regression
Age Prediction	**Right-Thalamus-Proper**	**Right-Thalamus-Proper**	**Left-Thalamus-Proper**	**Right-Putamen**
	Right-Accumbens-area	**Left-Putamen**	**Right-Putamen**	**Left-Thalamus-Proper**
	**Right-VentralDC**	**ctx-rh-precuneus**	**Right-Thalamus-Proper**	**ctx-rh-superiorfrontal**
	ctx-rh-medialorbitofrontal	**ctx-rh-superiorparietal**	Right-Caudate	**Right-Thalamus-Proper**
	**ctx-rh-precuneus**	**Right-Putamen**	Left-VentralDC	**Left-Putamen**
	**Left-Caudate**	ctx-rh-superiorfrontal	**Left-Putamen**	ctx-rh-superiorparietal
	**Right-Pallidum**	**Left-Caudate**	**ctx-lh-isthmuscingulate**	**ctx-lh-precuneus**
	**Left-Putamen**	**Right-VentralDC**	**ctx-rh-precuneus**	**ctx-rh-precuneus**
	Left-Hippocampus	Left-Thalamus-Proper	Left-Hippocampus	**Left-Caudate**
	**Left-VentralDC**	**ctx-lh-superiorparietal**	**Right-VentralDC**	**ctx-rh-insula**
	**ctx-rh-insula**	**Right-Pallidum**	**ctx-rh-superiorfrontal**	**ctx-lh-superiorfrontal**
	**ctx-rh-superiorparietal**	**Right-Caudate**	**ctx-rh-insula**	**ctx-lh-isthmuscingulate**
	ctx-lh-medialorbitofrontal	ctx-lh-precuneus	ctx-rh-isthmuscingulate	**Right-VentralDC**
	**Right-Caudate**	**Left-VentralDC**	**ctx-lh-superiorfrontal**	ctx-lh-superiortemporal
	**ctx-lh-superiortemporal**	**ctx-lh-superiortemporal**	**Left-Caudate**	ctx-rh-rostralmiddlefrontal
	Left-Pallidum	**ctx-rh-insula**	**ctx-lh-precuneus**	ctx-rh-middletemporal
	ctx-lh-middletemporal	ctx-lh-superiorfrontal	ctx-lh-insula	ctx-lh-middletemporal
	**ctx-lh-superiorparietal**	ctx-rh-inferiorparietal	Left-Pallidum	ctx-rh-inferiorparietal
	ctx-rh-isthmuscingulate	ctx-lh-isthmuscingulate	ctx-rh-parsopercularis	ctx-rh-precentral
	**Right-Putamen**	ctx-rh-superiortemporal	Right-Pallidum	ctx-rh-inferiortemporal
g-factor Prediction	**Right-Thalamus-Proper**	**Left-Putamen**	**ctx-rh-isthmuscingulate**	**Left-Thalamus-Proper**
	**Left-Putamen**	**Left-Thalamus-Proper**	**Right-VentralDC**	**Right-VentralDC**
	**Left-Hippocampus**	**Right-Putamen**	**ctx-rh-insula**	**ctx-rh-isthmuscingulate**
	**ctx-rh-superiorparietal**	**ctx-rh-superiorfrontal**	**Left-VentralDC**	ctx-lh-fusiform
	**ctx-rh-precuneus**	**Right-Thalamus-Proper**	**ctx-rh-inferiortemporal**	ctx-rh-caudalanteriorcingulate
	**ctx-lh-superiortemporal**	ctx-lh-superiorfrontal	Right-Caudate	ctx-lh-caudalanteriorcingulate
	**Left-Caudate**	**ctx-lh-superiorparietal**	ctx-lh-postcentral	**ctx-rh-lateraloccipital**
	ctx-lh-paracentral	**ctx-rh-superiorparietal**	**Right-Pallidum**	**Left-Pallidum**
	**ctx-lh-rostralmiddlefrontal**	**Left-Caudate**	**ctx-rh-lateraloccipital**	**ctx-rh-inferiortemporal**
	**ctx-lh-superiorparietal**	**ctx-lh-precuneus**	**Left-Pallidum**	**ctx-rh-insula**
	**ctx-lh-inferiorparietal**	**ctx-lh-inferiorparietal**	Left-Putamen	**Right-Pallidum**
	ctx-rh-parstriangularis	**Right-Caudate**	**ctx-rh-inferiorparietal**	**ctx-rh-fusiform**
	ctx-rh-isthmuscingulate	**ctx-rh-precuneus**	**ctx-rh-fusiform**	ctx-rh-posteriorcingulate
	**Right-Caudate**	**Left-Hippocampus**	ctx-rh-bankssts	**ctx-lh-isthmuscingulate**
	**ctx-lh-precuneus**	**ctx-rh-rostralmiddlefrontal**	ctx-rh-paracentral	**Left-VentralDC**
	**ctx-rh-superiorfrontal**	ctx-rh-inferiorparietal	**Left-Thalamus-Proper**	ctx-lh-precuneus
	ctx-rh-parsopercularis	**ctx-lh-rostralmiddlefrontal**	**ctx-lh-isthmuscingulate**	**ctx-rh-superiortemporal**
	**Left-Thalamus-Proper**	ctx-rh-lateraloccipital	**ctx-rh-superiortemporal**	ctx-rh-middletemporal
	**ctx-rh-rostralmiddlefrontal**	ctx-lh-precentral	ctx-lh-inferiorparietal	**ctx-rh-inferiorparietal**
	**Right-Putamen**	**ctx-lh-superiortemporal**	**Left-Hippocampus**	**Left-Hippocampus**
MHQ-factor Prediction	**ctx-lh-precuneus**	**Left-Putamen**	**Left-VentralDC**	**ctx-lh-posteriorcingulate**
	**Left-Putamen**	**Left-Thalamus-Proper**	**ctx-lh-posteriorcingulate**	**ctx-rh-isthmuscingulate**
	**ctx-rh-superiorparietal**	**Right-Putamen**	**ctx-rh-insula**	**Right-Pallidum**
	**ctx-rh-precuneus**	**ctx-rh-superiorparietal**	**ctx-lh-insula**	**Left-Pallidum**
	**ctx-lh-superiortemporal**	**Right-Thalamus-Proper**	**ctx-lh-middletemporal**	**Left-VentralDC**
	ctx-rh-superiortemporal	**Left-Caudate**	**ctx-rh-inferiortemporal**	ctx-rh-caudalanteriorcingulate
	ctx-lh-lingual	**ctx-lh-precuneus**	**ctx-rh-isthmuscingulate**	**ctx-rh-inferiortemporal**
	**Left-Caudate**	**ctx-rh-precuneus**	**ctx-lh-inferiorparietal**	**ctx-rh-insula**
	**ctx-lh-isthmuscingulate**	ctx-lh-superiorfrontal	**Right-Pallidum**	Right-Hippocampus
	**Right-Putamen**	**ctx-lh-superiorparietal**	**Left-Pallidum**	**ctx-rh-paracentral**
	**ctx-lh-fusiform**	**ctx-lh-isthmuscingulate**	ctx-rh-inferiorparietal	**ctx-rh-middletemporal**
	**ctx-lh-inferiortemporal**	ctx-rh-inferiorparietal	**ctx-rh-middletemporal**	**Right-VentralDC**
	Left-Hippocampus	**ctx-lh-superiortemporal**	**Right-VentralDC**	ctx-lh-lingual
	ctx-rh-lateralorbitofrontal	ctx-rh-superiorfrontal	ctx-rh-precuneus	**ctx-lh-inferiorparietal**
	ctx-lh-medialorbitofrontal	**ctx-lh-fusiform**	**ctx-lh-lateraloccipital**	**ctx-lh-lateraloccipital**
	**Right-Thalamus-Proper**	**ctx-lh-inferiortemporal**	**ctx-rh-lateraloccipital**	**ctx-lh-insula**
	**ctx-lh-superiorparietal**	ctx-lh-lateraloccipital	**ctx-rh-paracentral**	**ctx-lh-middletemporal**
	**Right-Caudate**	**Right-Caudate**	ctx-lh-parstriangularis	**ctx-lh-fusiform**
	**Left-Thalamus-Proper**	ctx-lh-inferiorparietal	**ctx-lh-fusiform**	ctx-rh-frontalpole
	ctx-lh-paracentral	ctx-lh-supramarginal	ctx-lh-medialorbitofrontal	**ctx-rh-lateraloccipital**

B.4.4 Training Progress of BrainNetCNN models for different prediction tasks based on six different dMRI measures

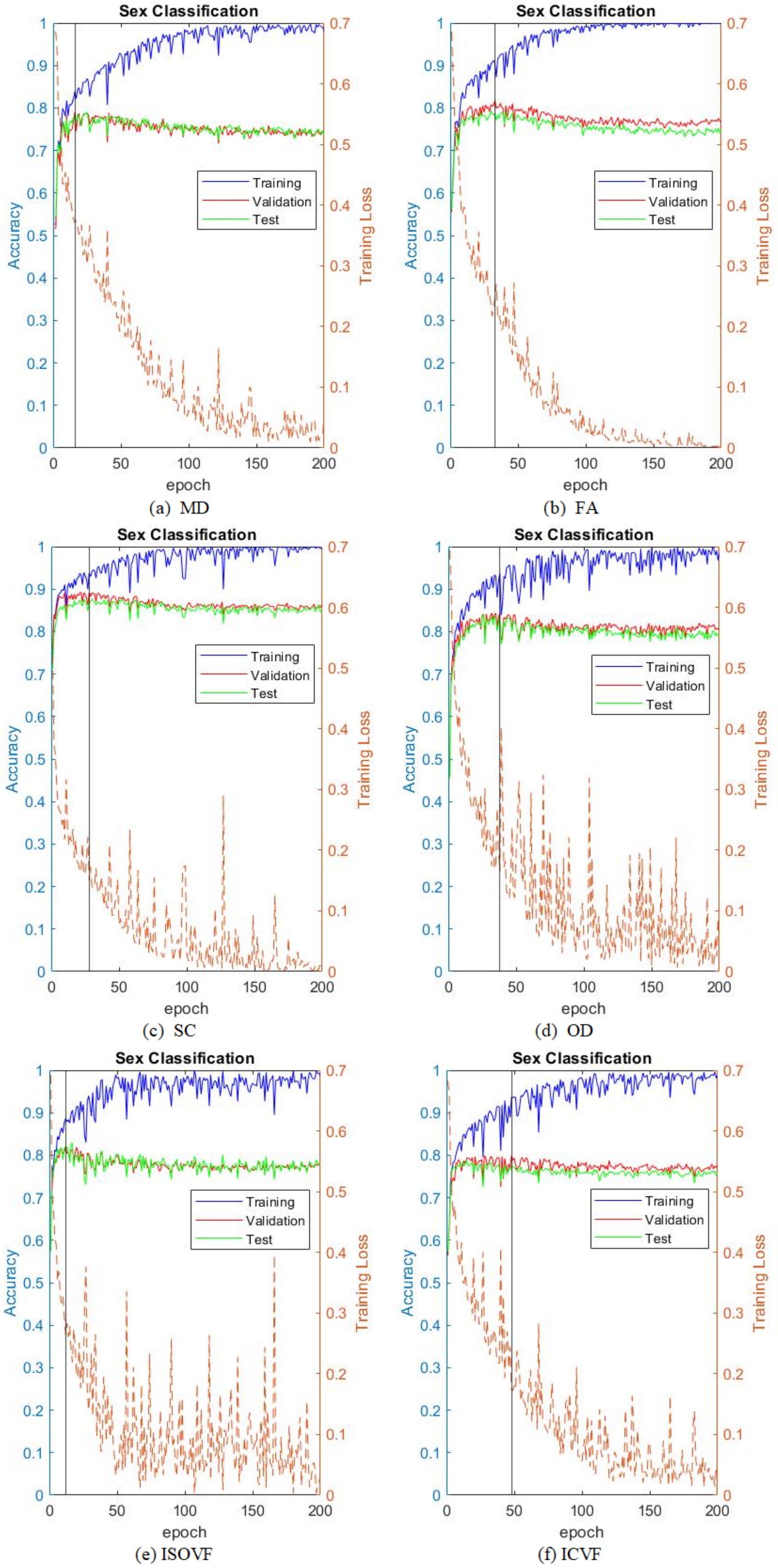

Figure 6: The BrainNetCNN models' training progress for sex classification for the fourth fold based on the six different dMRI measures. Training loss is the cross-entropy loss

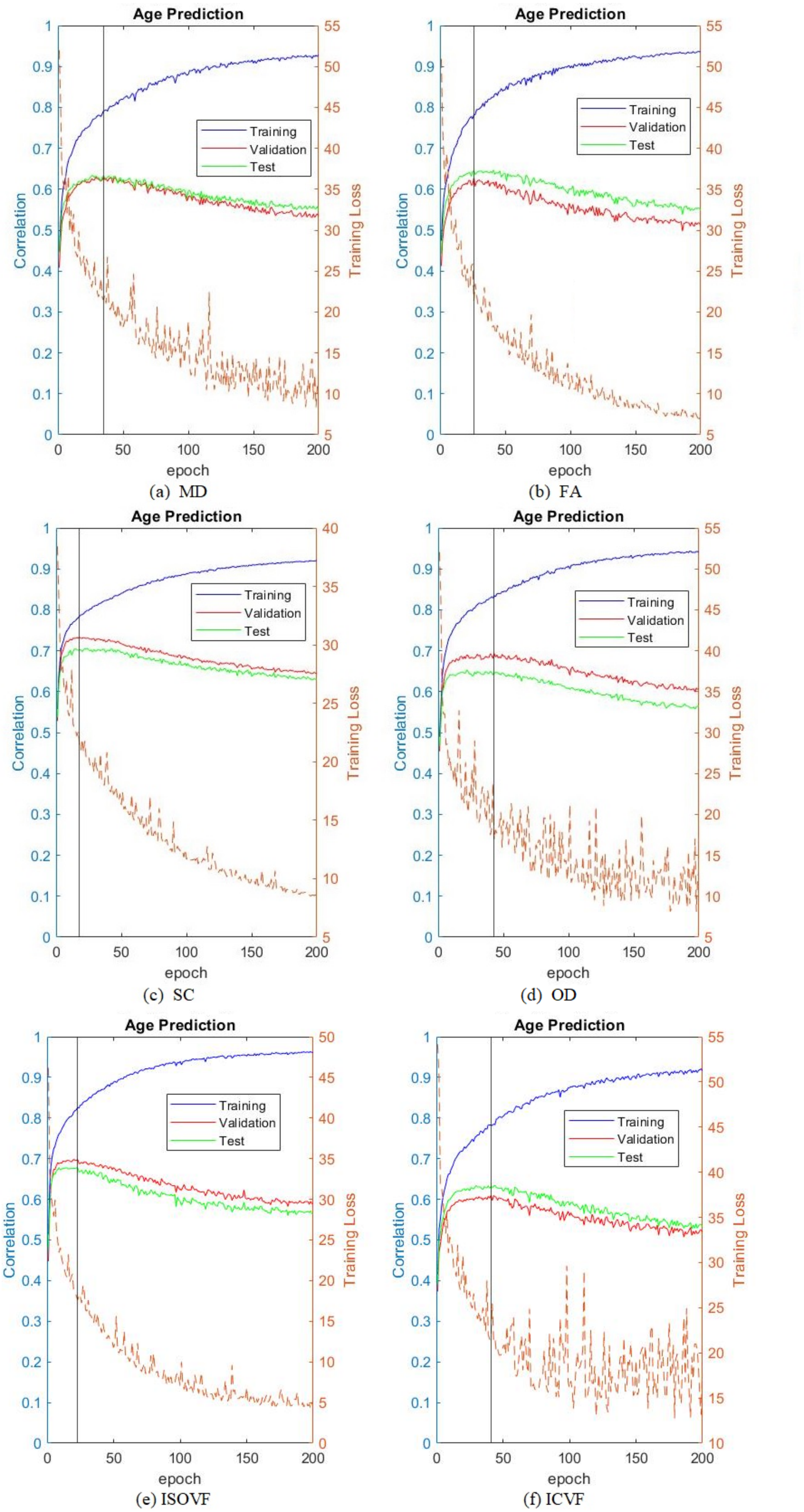

Figure 7: The BrainNetCNN models' training progress for age prediction for the fourth fold based on the six different dMRI measures. Training loss is the mean square loss

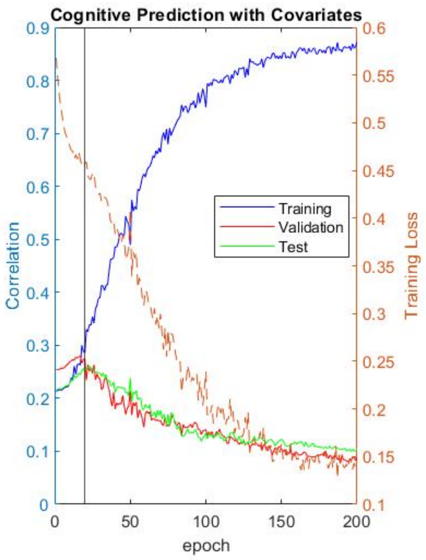

(a) MD

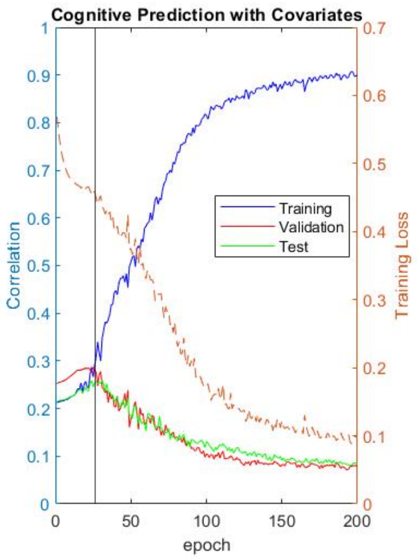

(b) FA

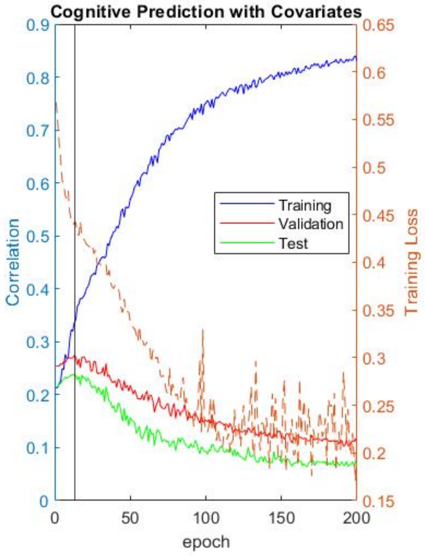

(c) SC

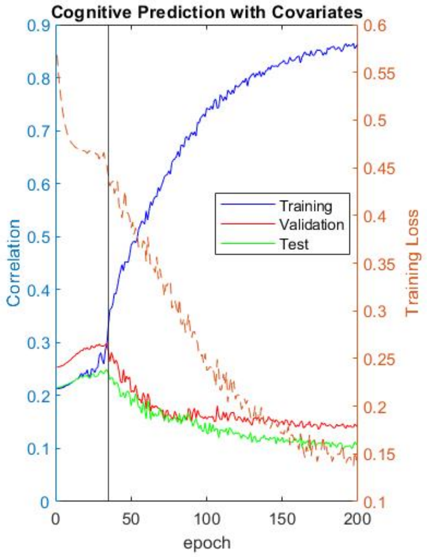

(d) OD

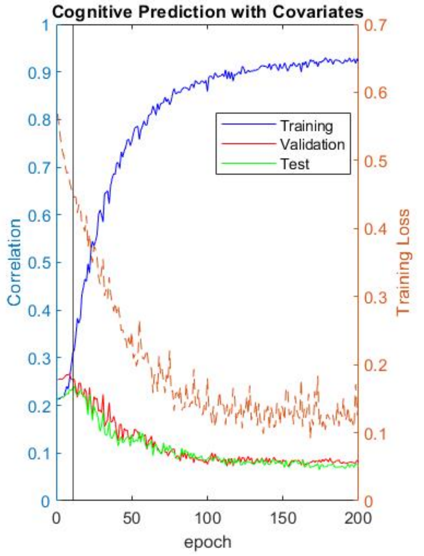

(e) ISOVF

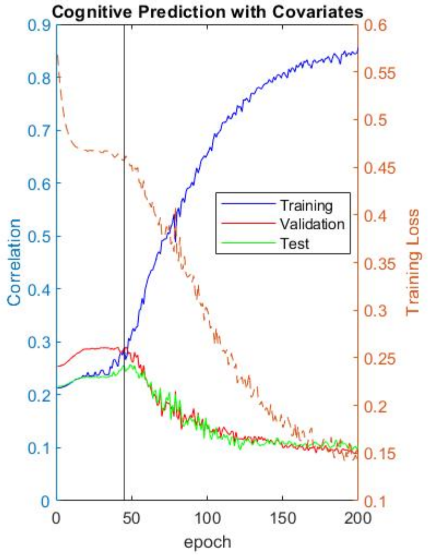

(f) ICVF

Figure 8: The BrainNetCNN models' training progress for g-factor prediction for the fourth fold based on the six different dMRI measures. Training loss is the cross-entropy loss

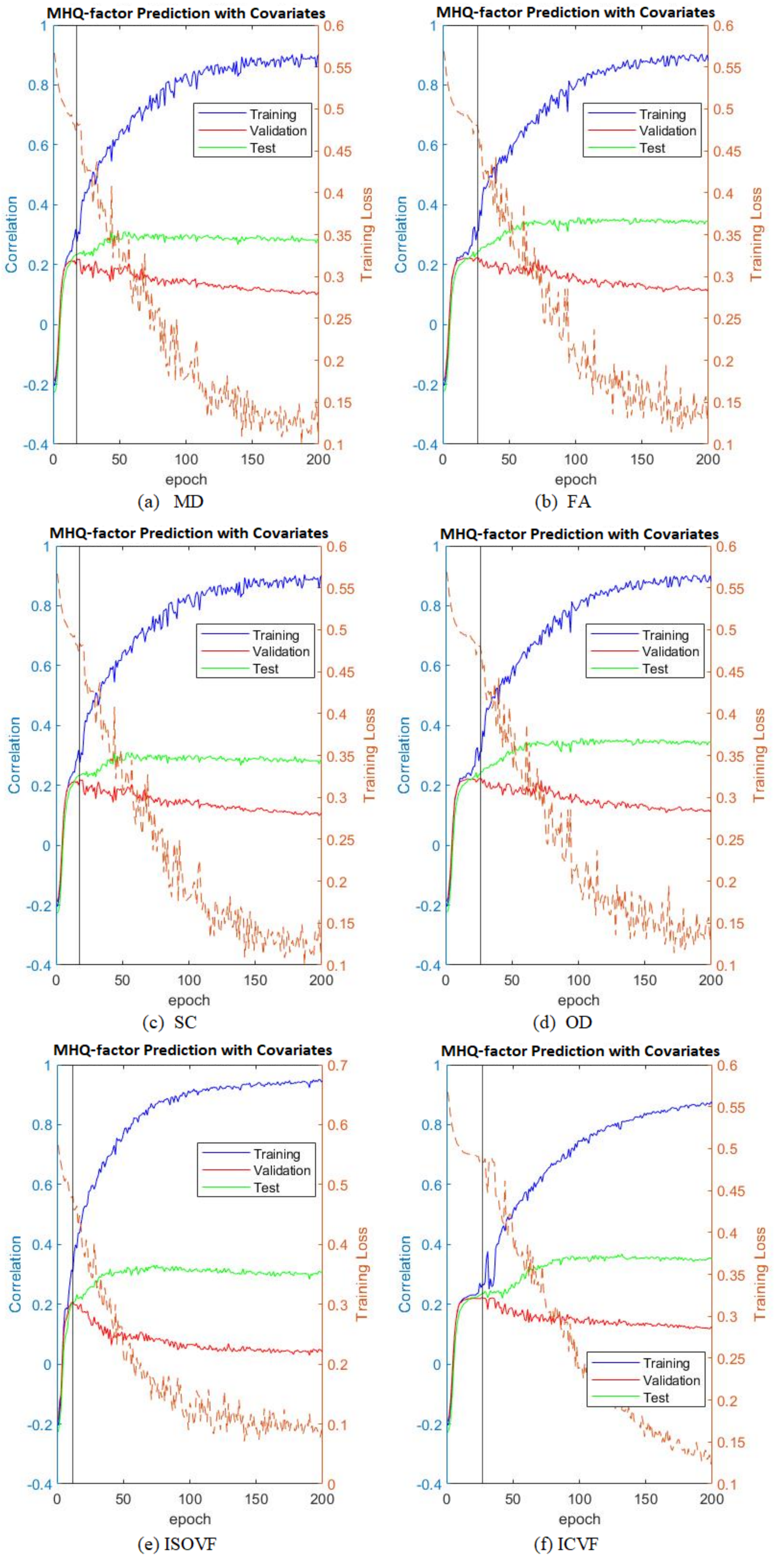

Figure 9: The BrainNetCNN models' training progress for MHQ-factor prediction for the fourth fold based on the six different dMRI measures. Training loss is the cross-entropy loss
